# Tackling Hominin Tickling: Bonobos Share the Social Features and Developmental Dynamics of Play Tickling With Humans

**DOI:** 10.1002/ajp.23723

**Published:** 2025-01-15

**Authors:** Elisa Demuru, Ilenia Montello, Jean‐Pascal Guéry, François Pellegrino, Florence Levréro, Ivan Norscia

**Affiliations:** ^1^ Laboratoire Dynamique du Langage, CNRS‐UMR 5596 Université de Lyon Lyon France; ^2^ ENES Bioacoustics Research Lab, CRNL University of Saint‐Etienne, CNRS, Inserm Saint‐Etienne France; ^3^ DBIOS Department of Life Sciences and Systems Biology University of Torino (DBIOS) Torino Italy; ^4^ La Vallée des Singes Romagne France; ^5^ Institut Universitaire de France Paris France

**Keywords:** emotion, gargalesis, intersubjectivity, ontogeny, tickling

## Abstract

It is under debate whether intersubjectivity—the capacity to experience a sense of *togetherness* around an action—is unique to humans. In humans, heavy tickling—a repeated body probing play that causes an automatic response including uncontrollable laughter (gargalesis)—has been linked to the emergence of intersubjectivity as it is aimed at making others laugh (self‐generated responses are inhibited), it is often asymmetrical (older to younger subjects), and it elicits agent‐dependent responses (pleasant/unpleasant depending on social bond). Intraspecific tickling and the related gargalesis response have been reported in humans, chimpanzees, and anecdotally in other great apes, potentially setting the line between hominids and other anthropoids. Here we investigated this phenomenon in bonobos and predicted that in this species (sharing with humans and chimpanzees the last common ancestor) the presence of tickling would be modulated depending on the players' age, play session initiators, and familiarity. In April–June 2018, we collected videos on play sessions—including tickling—on a bonobo group housed at *La Vallée des Singes* (France). We showed that tickling received decreased while tickling performed increased with age, with tickling being mostly directed from older to younger individuals. Moreover, tickling was mostly performed by the individuals that started the play interaction and most of it occurred in strongly bonded dyads, particularly mother–infant ones. Bonobo tickling features, especially age profile and social modulation, mirror those of heavy tickling in humans thus suggesting a common evolutionary origin and shared patterns of basic intersubjectivity in hominins.

## Summary

- We explored the ontogeny and social features of tickling play in bonobos.
- Bonobo tickling features mirror those observed in humans.
- We suggest that tickling can be used as a behavioral marker to investigate the biological bases of intersubjectivity within the hominid family.

## Graphical abstract

Graphical abstract showing the main results of this article.

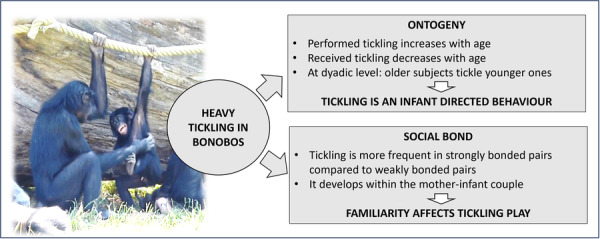

## 1 Introduction

Intersubjectivity can be broadly defined as the capacity to share and represent other's inner states, including emotions, desires, attentional foci, intentions, and beliefs allowing two subjects to experience a sense of *togetherness* around a certain action (Gärdenfors 2007). From an historical and philosophical point of view, intersubjectivity has been considered as a language‐related capacity, thus drawing a clearcut separation between humans and nonhumans. Although it is undeniable that language and certain forms of intersubjectivity are linked, it would be a mistake to limit intersubjectivity to those complex human skills (Zlatev 2008). In the last decades, a much more nuanced picture has emerged: intersubjectivity should be viewed as a multilayered phenomenon, with complex socio‐cognitive capacities emerging on more automatic, socio‐emotional ones that are found at the core (Zlatev, Persson, and Gärdenfors 2005; Preston and de Waal 2002). Such a framework incites to adopt a bottom‐up perspective and explore if and to what extent intersubjective capacities might be found in species other than humans (de Waal and Ferrari 2010).

In humans, a behavior that has been linked to the emergence of intersubjectivity is heavy tickling (here after, tickling), a playful pattern that induces uncontrollable laughter or “gargalesis” (as opposed to “knismesis,” the unpleasant sensation elicited by a light touch across the skin; Hall and Allin 1897; Harris 1999). Indeed, the implicit ability to discriminate between self and others' generated stimulation is an essential building block of primary intersubjectivity (Stevanovic and Koski 2018) and a crucial element of gargalesis is that it cannot be self‐induced but requires external triggers (Harris 1999). Humans are unable to tickle themselves possibly due to a prediction mechanism that anticipates the sensations deriving from self‐initiated movements and inhibits self‐generated responses (Blakemore, Wolpert, and Frith 2000). Another element is that tickling in humans appears to be intentionally performed to make another individual laugh (Harris 2012). In this respect, tickling success depends on a common, joint action where the agent that delivers tickling receives a positive reinforcement feedback promoted by gargalesis (Harris 2012; Provine 2004). Therefore, tickling involves a type of intersubjectivity and its investigation can help understand whether intersubjectivity abilities set the line between humans and nonhuman primates (Stevanovic and Koski 2018).

Via a heavy, repetitive pressure on specific body areas, tickling also induces a set of behavioral responses: withdrawing from the tickling actor, sheltering the ticklish areas, and wriggling (Harris 2012; Leuba 1941; Selden 2004). Gargalesis could be an implicit, low‐level response (Black 1984; Hall and Allin 1897; Harris 1999; Harris and Christenfeld 1999) that may be socially modulated, as some automatic processes are amplified by certain affective contexts (Bradley, Lang, and Cuthbert 1993) or strong social bonds (Palagi et al. 2020). The body of literature focusing on emotional contagion is a good example of how automatic responses—such as yawn contagion (*Homo sapiens*, Norscia & Palagi 2011; *Pan troglodytes*, Campbell and de Waal 2011), rapid facial mimicry (Mancini, Ferrari, and Palagi 2013), pupil dilatation (*Homo sapiens*, Kret, Fischer, and De Dreu 2015)—are affected by social affiliation, with individuals responding more frequently to familiar others and in‐group members than with strangers.

Similarly, the basic tickling response involving laughter is thought to be automatic and the whole set of behavioral responses to tickling might have a communicative function (Provine 2004). In humans, tickling may be adaptive in nurturing mother–infant bond through play (Harris 2012; Leuba 1941; Provine 2004). Moreover, a further adaptive value of tickling may reside in the gap between the negative inward sensation and the positive outward signals of the tickled subject (Harris 2012). Laughter might encourage others' tickling whereas the unpleasant sensation might support the development of combat skills through the promotion of play‐fighting (Harris 2012). A further element is that tickling ontogeny in children closely follows the development of intersubjectivity (Bard et al. 2014; Steinbeis 2016). Tickling‐induced laugher appears later than spontaneous laughter in human ontogeny (Ishijima and Negayama 2017). Tickling is asymmetrical, in that it is first received (by immature subjects), later requested to others, and then delivered to others (as individuals grow old; Bard et al. 2014; Cochet and Vauclair 2010; Crais, Watson, and Baranek 2009; Leavens and Bard 2016). Finally, the response to tickling may vary depending on the agent that delivers the tickle. Washburn (1929) reported that infants may respond with cries (or show no response) to tickling performed by strangers and with laughter to tickling performed by their parents. In this respect, the same physical stimuli can evoke opposite reactions (laughter or fear) depending on social variables (Rothbart 1973).

Tickling has been used as an experimental stimulation in the framework of understanding the evolutionary origins of human laughter. It has been demonstrated that juveniles of all the great ape species (i.e., orangutans, gorillas, chimpanzees, and bonobos) produce play‐context specific panting vocalizations when tickled by humans (Davila Ross, Owren, and Zimmermann 2009). Interestingly, inter‐specific human tickling has been shown to cause specific behavioral and acoustical features in a much more distantly related species: the rat (*Rattus norvegicus*). Rats that emit high‐frequency ultrasonic chirping while playing with conspecifics, but such play‐induced vocalization is also produced when humans vigorously stimulate rats in specific body areas (Panksepp and Burgdorf 2010). It is worth noting that this study also showed that such response is higher in young rats compared with adult rats and that it is inhibited in aversive environmental conditions.

Intraspecific tickling has only been described in extant hominids, as it has been observed in orangutans, gorillas, and the *Pan* genus (chimpanzees and bonobos) during play‐fighting (Harris 2012). Therefore, tickling is a good behavioral mark to investigate whether the distinction between self and others is present in hominids—other than humans—in the context of emotional communication, and to study the developmental trajectory in our closest living relatives. Yet, despite its relevance to the understanding of the evolution of human intersubjectivity, tickling has received scarce attention in nonhuman apes, although some qualitative studies, reported below, described some features of tickling in chimpanzees. When tickled, nonhuman great apes show the equivalent of human laughter with an open mouth display (typically the full play face, i.e., both the upper and lower teeth are visible) associated with a breathy vocalization, named panting laugh (Hooff and Preuschoft 2003; Davila Ross, Owren, and Zimmermann 2009). As in humans, also in chimpanzees, tickling play has been especially observed between mothers and infants where laughter can elicit the mother's attention and serve as a nonverbal communicative tool for the infant (Provine 2004). Similar to humans, chimpanzee (*Pan troglodytes*) infants have been reported to initially respond to tickling by sheltering the tickled area and, over time, they start to exhibit this behavior as a tickle anticipation (Bard et al. 2014). At about 1 year old, chimpanzees intentionally request tickling by gestures that consist in sheltering their body or bringing the mother's hands to the desired tickled area while performing a smile (Bard et al. 2014). Besides this early tickle response and solicitation, infant chimpanzees do not actively tickle others until they are older, during juvenility (Leavens 2009; Leavens and Bard 2016). To our knowledge, no study so far has quantitatively investigated tickling dynamics in nonhuman great apes. This study aims to explore tickling ontogenesis and social modulation in a group of bonobos (*Pan paniscus*) by analyzing dyadic play. As in chimpanzees, the development of bonobo infants is slow and immatures' care is provided exclusively by the mother (Kano 1992). This species shows great social tolerance and high playfulness (Gruber and Clay 2016), both in the wild (Behncke 2015) and in captivity (Demuru, Ferrari, and Palagi 2015; Palagi 2006). The presence of players belonging to different age classes, high frequency of social play, and high levels of social tolerance make bonobos a suitable model species to study the ontogeny and social‐related characteristics of tickling.

Based on the previous framework and on the phylogenetic closeness between bonobos and other hominins (humans and chimpanzees) we formulated the following predictions on how ontogeny, play initiators and recipients, and the familiarity of the tickler may affect tickling dynamics in bonobos.

### 1.1 Prediction 1—Developmental Trajectory

Eleven‐month‐old toddlers, as well as 1‐year‐old chimpanzees, consider others as possible triggers for their emotional responses and intentionally request to be tickled (Bard et al. 2014; Cochet and Vauclair 2010; Crais, Watson, and Baranek 2009). Only when they are older, during juvenility, chimpanzees start to actively tickle others (Leavens and Bard 2016). Indeed, in both humans and chimpanzees, tickling appears to be an adult/subadult behavior toward younger individuals (Bard et al. 2014; Leavens and Bard 2016). If similar situation applies to bonobos, we expect (i) a positive correlation between the tickler's age and the time spent tickling (*prediction 1a*); (ii) that the time an individual is tickled be negatively correlated with the individual's age (*prediction 1b*); (iii) that older players perform more tickling on younger players compared to younger on older players or same age players (*prediction 1c*).

### 1.2 Prediction 2—Play Initiation in Relation to Tickling

In humans, tickling is performed by an agent during play to make another individual laugh, with positive feedback enhanced by the gargalesis response of the other (Harris 2012). Hence, individuals may intentionally initiate a playful interaction to then start a tickling session. If the same applies to bonobos, we expect that the individuals who initiate play are also most likely to initiate tickling during the session.

### 1.3 Prediction 3—Familiarity Effect

It has been shown that bonobos respond more frequently to familiar partners in vocal turn‐taking interactions and therefore familiarity plays an important role in the choice of interlocutors (Levréro et al. 2019). In humans, to elicit a pleasurable sensation and laughter, tickling must come from individuals that are familiar and liked (Harris 2012; Rothbart 1973). To our knowledge, no study so far has investigated this aspect in apes. If a familiarity effect applies to bonobos, we expect strongly bonded dyads‐ and especially mother–infant dyads—to spend more time in tickling interactions compared to weakly bonded ones.

## 2 Materials and Methods

### 2.1 Study Site and Group

The studied bonobo group was housed at *La Vallée des Singes* (Romagne, France) and observed for 3 months (April–June 2018). The bonobo facility included an indoor space (~500 m^2^) and a wooded external island (~1 ha). The group was composed of 17 individuals (age range: 1–50 years ± 12.55; adults, age ≥ 11 years: three males and six females; juveniles 6–10 years: two males and two females; infants, 0–5 years: one male and three females; see Table S1 for group composition). Feeding sessions took place four times per day: 11:30 a.m., 2:30 p.m., 3:45 p.m., and 5:00 p.m. Animals were provided with vegetables, fruit, and primate dry food. Water was available ad libitum, and several environmental enrichments were provided (lianas, trunks, platforms). No aberrant behavior was observed during the study period.

Observations were performed from 9:30 a.m. to around 6:30 p.m. Six days a week when the animals were in the outside enclosure. Scan sampling (every 3 min for an individual mean of 2024.12 ± 202.36 scans/per ind.) was used to collect data on grooming sessions to establish strongly and weakly bonded dyads. Data were collected through continuous video recordings by applying focus group sampling (Altmann 1974). Play session data were collected by using the all occurrences sampling method (Altmann 1974). A total of 1052 play sessions were recorded, including polyadic and dyadic play sessions. For each play session, players' identities, age, sex, identity of the individual that started the play session, play session duration, and whether or not the session included tickling were recorded. ED trained IM to recognize tickling sessions until the level of interobserver reliability was > 85%. Then, a single researcher (I.M.) performed video coding by using the free software PotPlayer and by reporting the information on Microsoft Excel spreadsheets.

### 2.2 Operational Definition and Statistics

Dyadic and polyadic play sessions were recorded. Given the complex dynamics characterizing polyadic sessions (*N* = 272), only dyadic play was considered for this study. We decided to keep in the analyses only those dyadic play sessions that lasted at least 10 s and were recorded from the beginning to the end of the play session (*N* = 564), for example, we excluded those sessions during which the animals went out of sight. We decided to set a lower limit of play duration as sessions lasting few seconds were rare and basically consisted of repeated play invitations with a very limited exchange of play patterns. A play session started when a group member invited another one to play by performing a directional play pattern that initiated the playful interaction. The session ended when the two players ceased their activity, one of the two left or there was a third member intervention (who interrupted the session, substituted one of the players, or joined the play session). If the play started again by the same two players within a 30 s interval after the two stopped playing, it was considered as a pause of the same play session. Play sessions were examined to determine play duration, players identity, and tickling occurrences (actor, receiver, and duration). Tickling was reported when there was a repetitive, repeated grasping pressure of the hands/feet/mouth on specific body areas, that is, the neck/belly/feet/armpits. A tickling bout started when the actor contacted the tickled body part and ended when the contact was over (example of tickling session: Video S1). Due to the repetitive nature of tickling, a single tickling session was reported when more tickling bouts occurred closely together, that is, less than 5 s between the end of one tickling bout and the beginning of the following one. To our knowledge, no study has ever characterized the time between tickling bouts to establish an interval, so we established a priori a threshold of 5 s.

Affiliation was calculated using the grooming behavior collected through scan samples. Dyads whose social bonding score (number of scans in which there was grooming on the total number of scans where the two animals were visible) was in the upper quartile (25% of data distribution) were categorized as strongly bonded (friends/mother‐offspring), the others were considered to have a weak affiliative relationship (this category includes dyads that groomed a little and dyads that did not show grooming interactions). Subjects' kinship was known (Table S1).

### 2.3 Statistical Analysis

We checked data normality through the Kolmogorov–Smirnov test before running all statistical tests. When the condition was violated (*p* < 0.05), we employed nonparametric tests. Analyses were two‐tailed, and the significance level was set at 5%. We used the Spearman's test to check for correlation between (i) actor's age and tickling duration on actor's total play duration; (ii) receiver's age and time spent being tickled on receiver's total play time. We used the Pearson's test to check for a correlation between indegree centrality values (see below for an explanation) in social play with tickling and without tickling. We used the Kruskal–Wallis's test to check directionality at dyadic level according to three different age classes (infants: ≤ 5 years old; juveniles: 6–10 years old; adults: > 10 years old) so that we could compare three categories: same age class (immature‐immature; juvenile‐juvenile; adult‐adult), older to younger (adult‐juvenile; adult‐immature; juvenile‐immature) and younger to older (immature‐juvenile; immature‐adult; juvenile‐adult). We used the Mann–Whitney test to compare the time spent tickling on total play time for two categories: strongly‐ and weakly‐bonded individuals. We used the Wilcoxon signed rank test to check for a link between the directionality of play invitation and tickling, and the correlation between clustering coefficients in the social networks of social play with and without tickling (see below for definitions).

We obtained the social networks (measures and representations) of play with and without tickling by using the freeware Gephi 0.9.7 (www.gephi.org/; dual license CDDL 1.0 and GNU General Public License v3). The network is composed of individuals (nodes) and interindividual relations (directed edges) derived from the proportion of dyadic directional interactions (AB if A was the initiator and B the recipient of the interaction; BA if the other way around). We calculated the proportion as the number of play invitations over the dyadic play time for the networks of play either with or without tickling. From play (with and without tickling networks) we extracted, for correlation, the clustering coefficient. We also extracted modularity and indegree centrality values from the social network graphs. For a given node, its (local) clustering coefficient represents the fraction of existing over possible links within its neighbors (Watts and Strogatz 1998; Bhattacharya et al. 2023). Modularity refers to the number of edges falling within groups minus the expected number in an equivalent network with edges placed at random (Newman 2006). Indegree centrality refers to the number of edges directed toward each node (Bringmann et al. 2019).

## 3 Results

**Figure 1:**
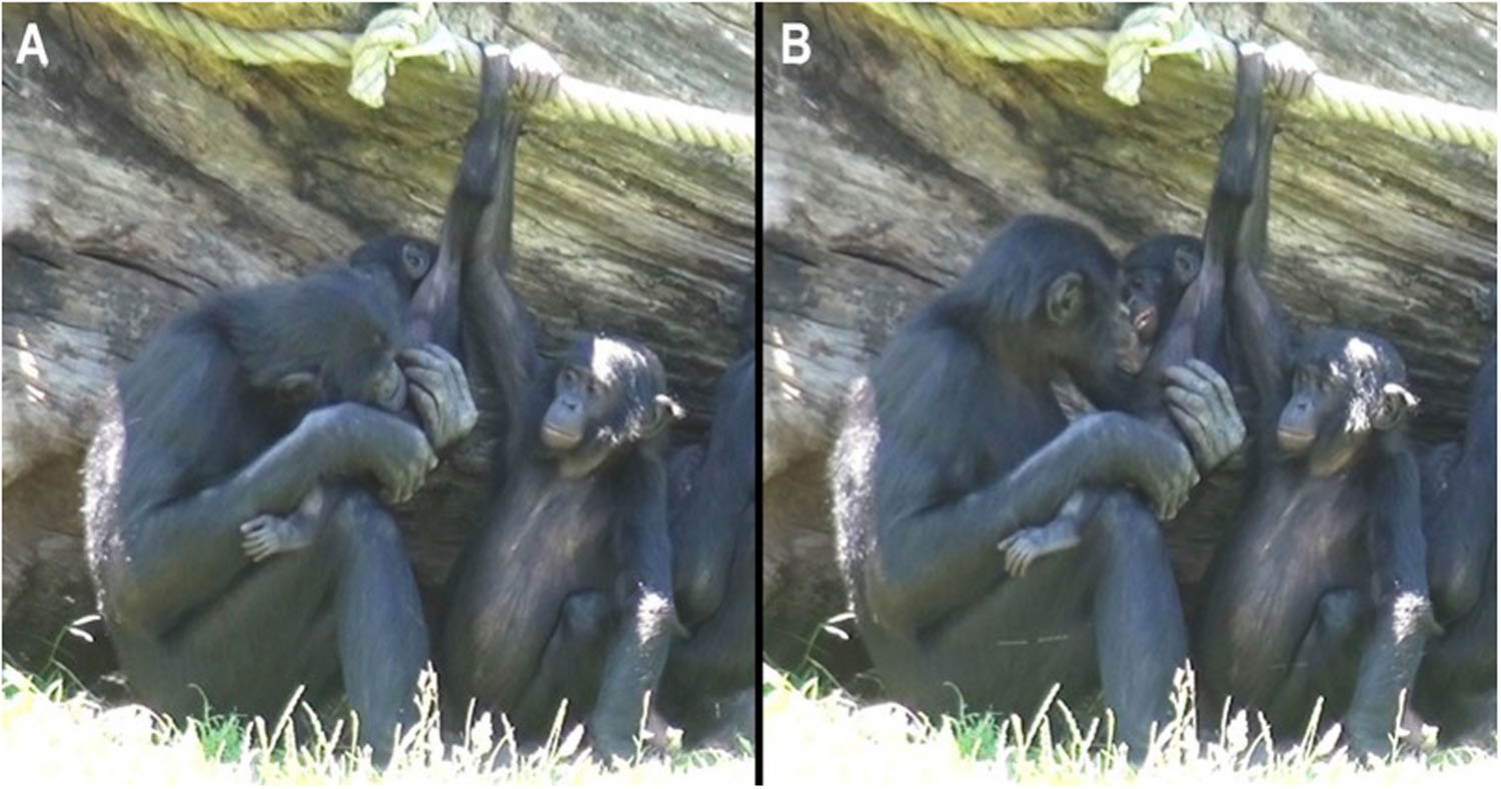
Play tickling sequence. An adult male bonobo tickles an infant female bonobo by using its mouth (A). The male checks the face of the infant right after the tickling sequence (B).

A total of 564 play sessions were examined (*N*_tickling _= 75; i.e., 13.3% of total play sessions): 195 involved two immatures (*N*_tickling_ = 11; i.e., 5.6%), 97 involved two juveniles (*N*_tickling _= 17; i.e., 17.5%), 196 involved an immature and a juvenile (*N*_tickling_ = 34; i.e., 17.3%), 56 involved an immature and an adult (*N*_tickling_ = 7; i.e., 12.5%), and 20 involved a juvenile and an adult (*N*_tickling_ = 6; i.e., 30.0%). A total of 14 individuals were observed performing tickling (actors) and a total of nine individuals were observed receiving tickling (receivers). One subject, a 1‐year‐old infant, was only seen receiving tickling, six individuals were only actors of tickling, eight individuals were both actors and receivers and two 17‐year‐old individuals never performed nor received tickling (see Table S1 for details). No adult–adult play session was recorded. Figure 1 and Video S1 show a tickling sequence.

### 3.1 Age Effect

**Figure 2:**
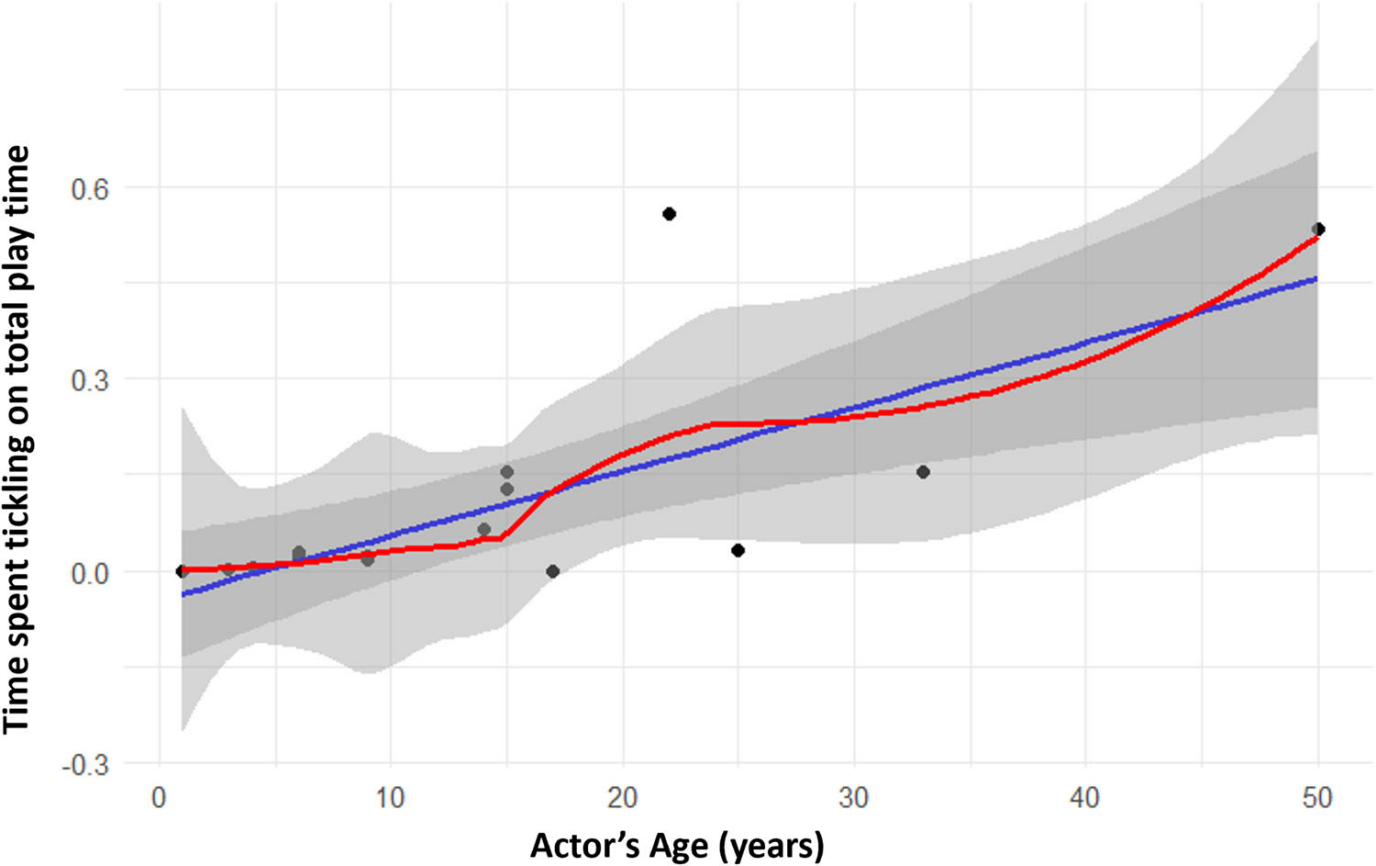
Scatter plot showing the relationship between actor's age and time spent tickling on total play time. Points: data value distribution; lines: regression line (blue) and LOESS curve (red); bands: confidence interval (0.95). The correlation is significant.

**Figure 3:**
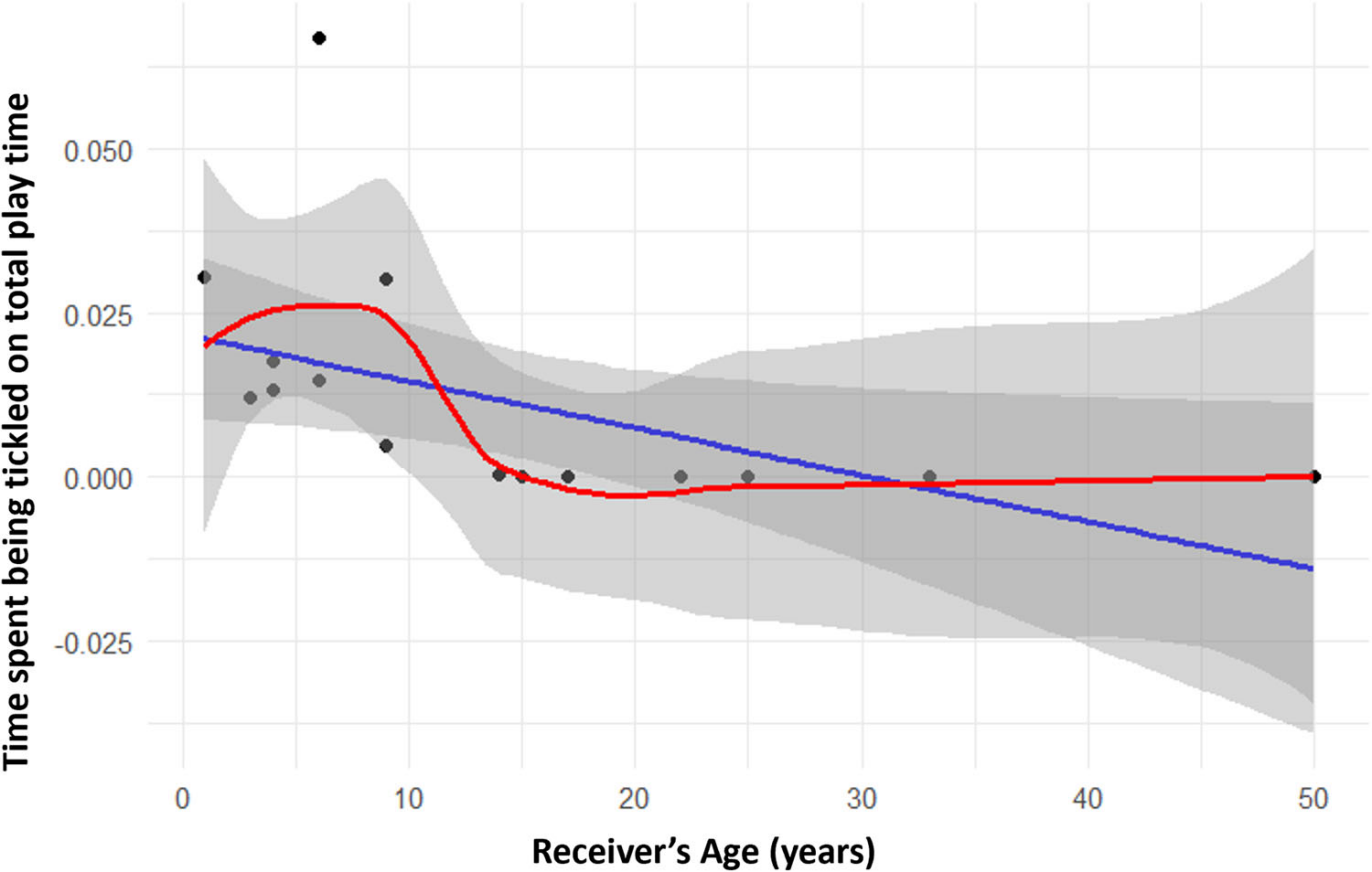
Scatter plot showing the relationship between receiver's age and time spent being tickled on total play time. Points: data value distribution; lines: regression line (blue) and LOESS curve (red); bands: confidence interval (0.95). The correlation is significant.

**Figure 4:**
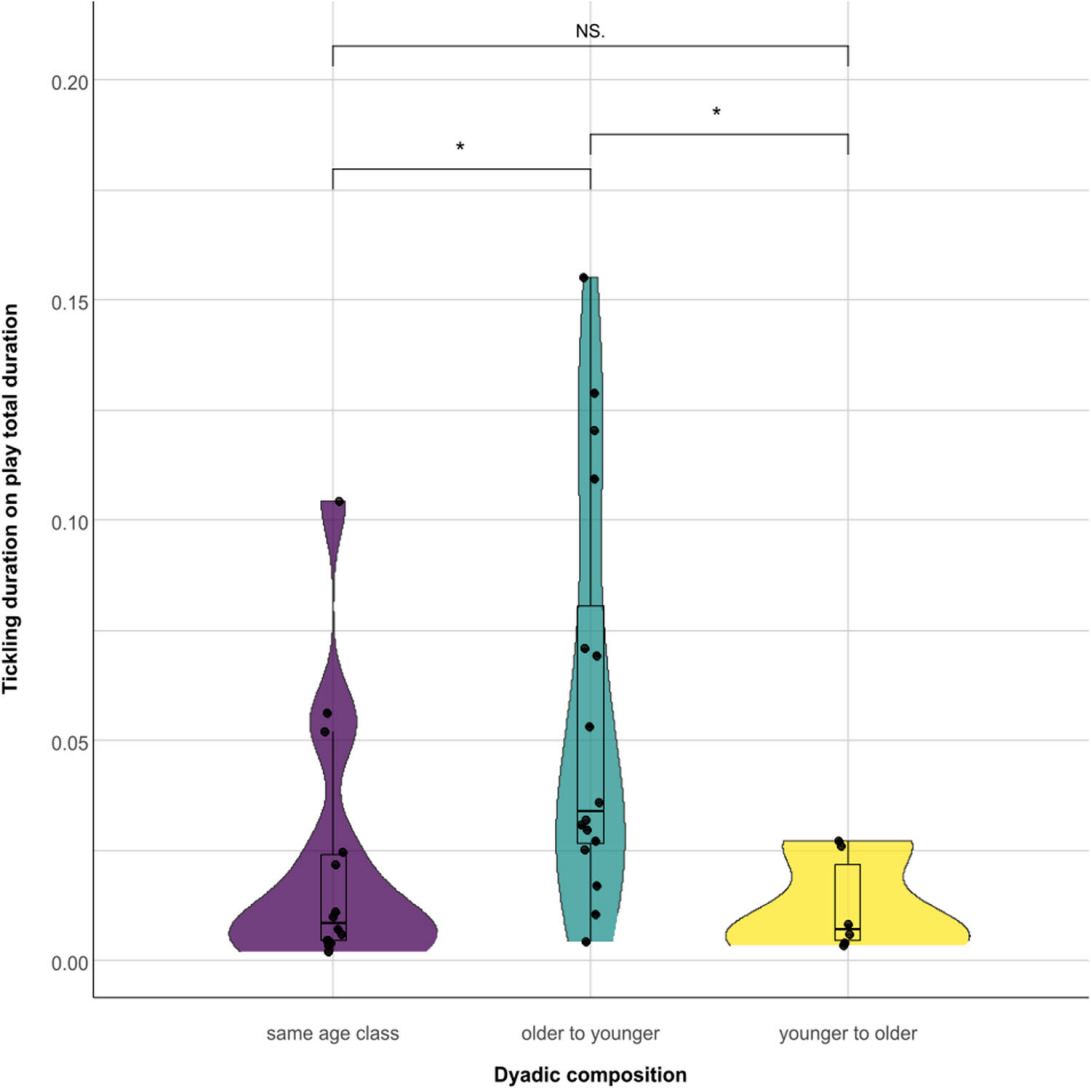
Tickling duration on play total duration in three different dyadic compositions: the tickler is in the same age class as the ticklee, the tickler is older than the ticklee and the tickler is younger than the ticklee. The violin plot and the points show the distribution of the data, the nested box plot shows some summary statistics (Horizontal line: median; box length: interquartile range; vertical line: minimum and maximum values in the data). NS = nonsignificant; * = *p* < 0.05. Tukey post‐hoc test: same age class vs. older to younger *p* = 0.019; same age class vs. younger to older *p *= NS; younger to older vs. older to younger *p* = 0.019.

We first explored whether tickling behavior follows an ontogenetic trajectory both on the actor and receiver's side. Results show a positive significant correlation between actor's age and time spent tickling on actor's total play time (Spearman's test: *N*_individuals_ = 17; *r*_s_ = 0.609; *p* = 0.010; Figure 2). As for receivers, we found a negative significant correlation between receiver's age and time spent being tickled on receiver's total play time (Spearman's: *N*_individuals_ = 17; *r*_s_ = –0.853; *p* = 0.039; Figure 3). We checked tickling directionality at the dyadic level according to three different age classes (immature, juvenile, adult). The duration of tickling as a proportion of total play time was found to be statistically different according to dyadic age composition (same age class, older to younger, younger to older). Results show that the “older to younger” category dyads showed significantly higher frequencies of tickling compared with the other two categories (nonparametric test for *k*‐independent samples: *N* = 37; *p* = 0.007; Tukey post‐hoc test: same age class vs. older to younger *p* = 0.019; same age class vs. younger to older *p* = NS; younger to older vs. older to younger *p* = 0.019; Figure 4).

### 3.2 Play Invitation and Tickling

**Figure 5:**
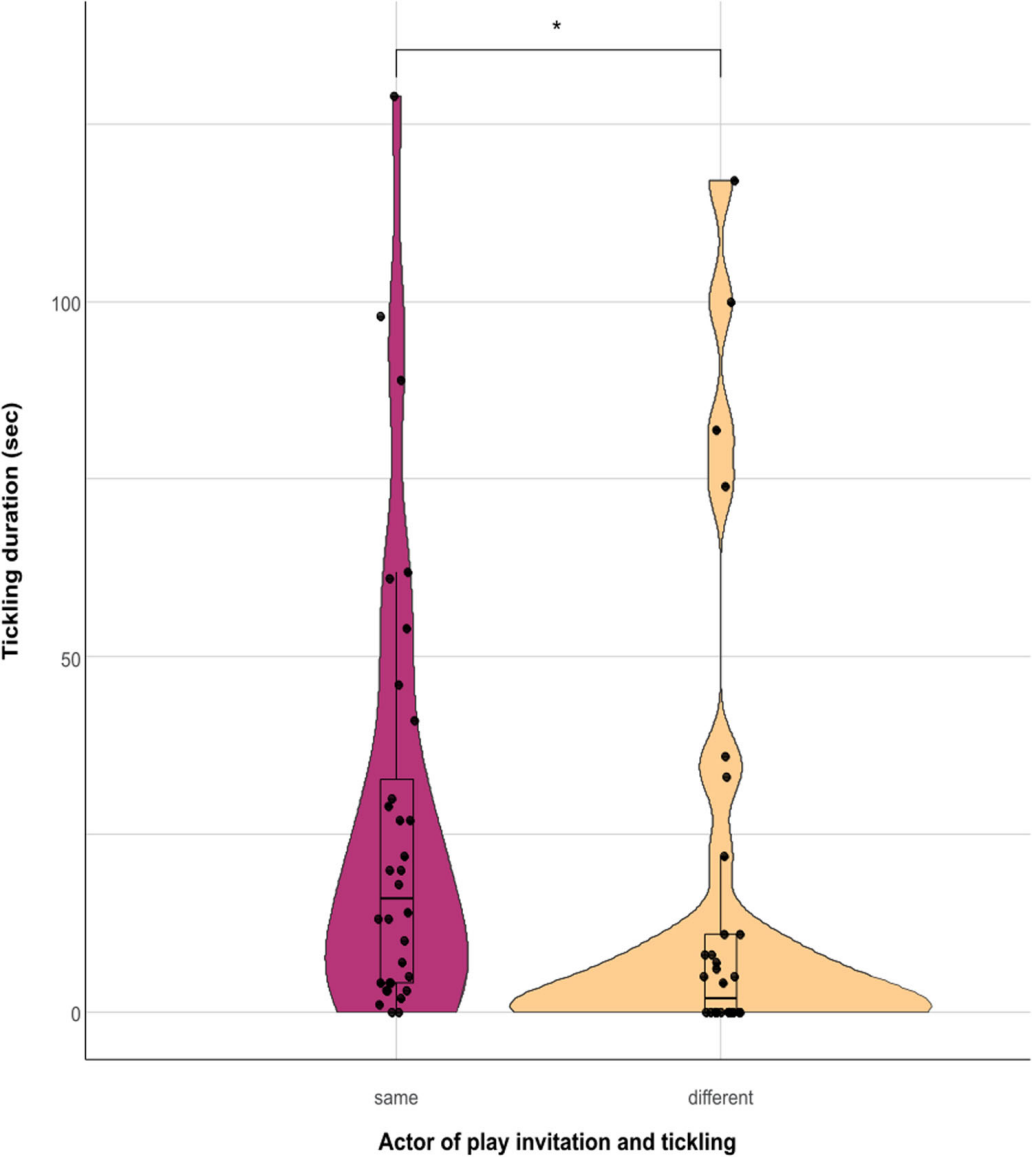
Difference in tickling duration when the subject that performed tickling is the one that started the play session (same) and when it is the one that accepted the play invitation (different) (Wilcoxon signed rank test, with Monte Carlo correction (10.000 permutations), *N*_dyads_ = 32; *Z* = –2.169; *p* = 0.029). The violin plot and the points show the distribution of the data, the nested box plot shows some summary statistics (Horizontal line: median; box length: interquartile range; vertical line: minimum and maximum values in the data). * = *p* < 0.05.

We investigated a possible link between play invitation and tickling performed. We found that the time an individual spent tickling was significantly higher when it started the play session compared to when it accepted to play after receiving a play invitation (Wilcoxon signed rank test, with Monte Carlo correction (10.000 permutations), *N*_dyads_ = 32; *Z* = –2.169; *p* = 0.029; Figure 5).

### 3.3 Social Bond

**Figure 6:**
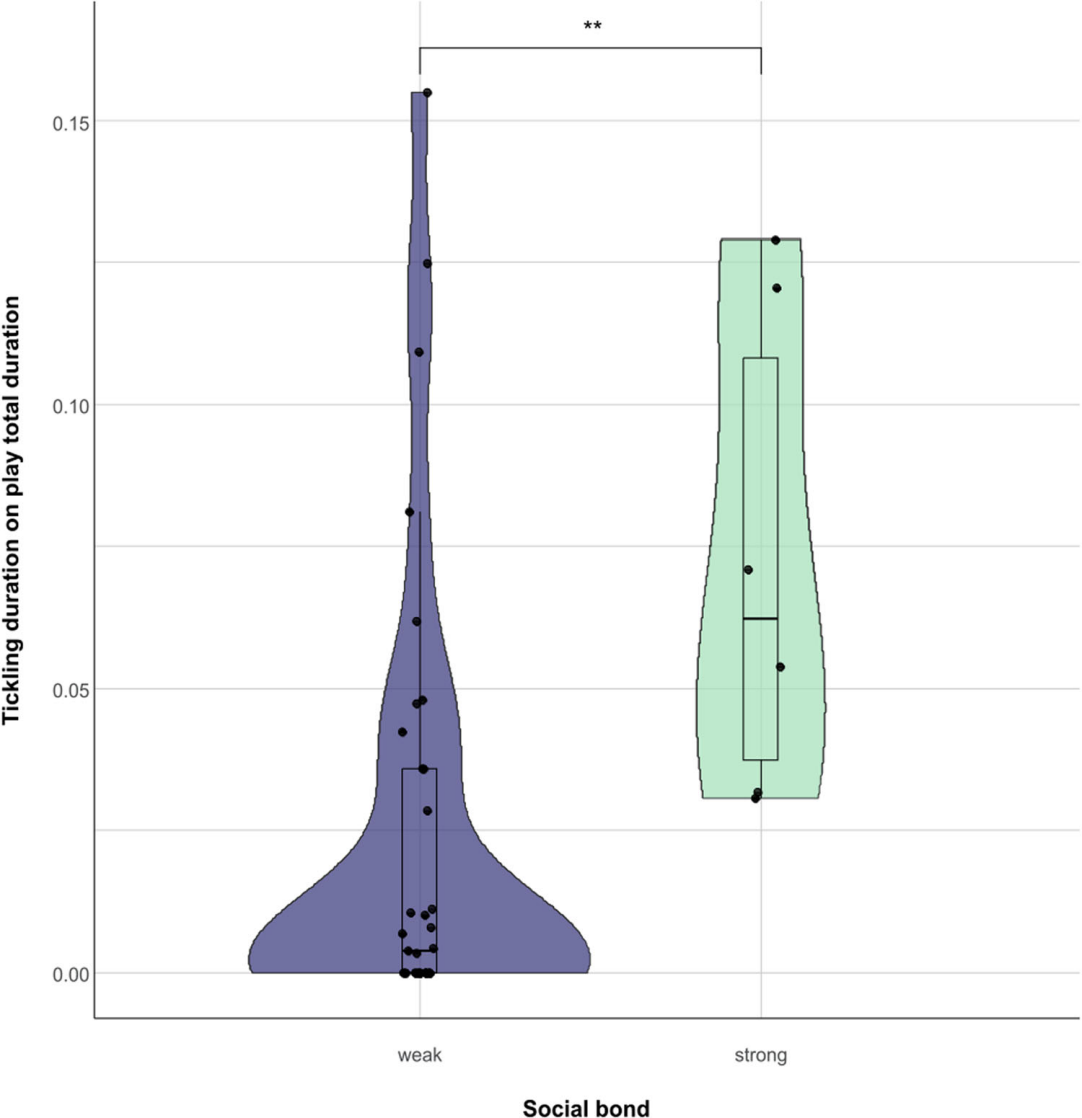
Difference between tickling duration on play total duration in weakly and strongly bonded dyads (Mann Whitney *U* = 32.000; *N*_strong_ = 6; *N*_weak_ = 35; *p* = 0.006). The violin plot and the points show the distribution of the data, the nested box plot shows some summary statistics (Horizontal line: median; box length: interquartile range; vertical line: minimum and maximum values in the data). ** = *p* < 0.01.

**Figure 7:**
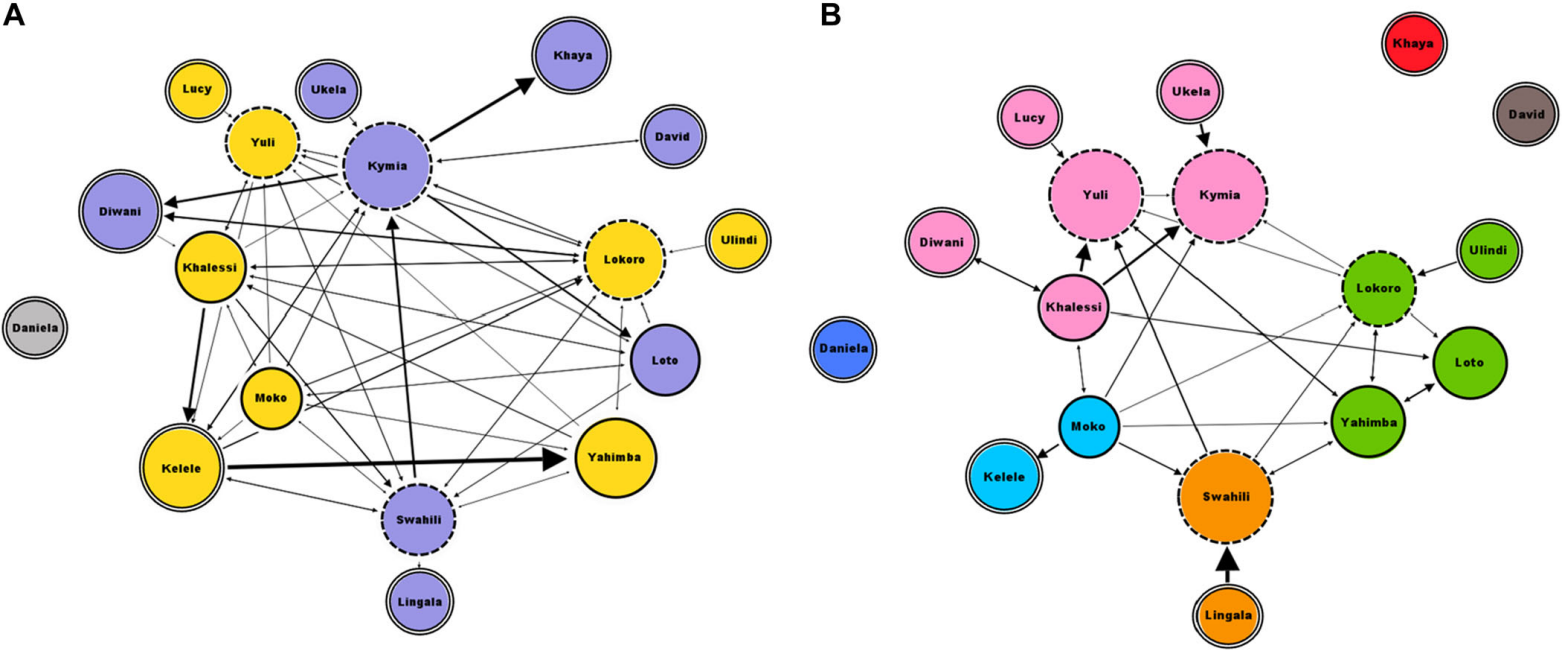
Social networks of social play without tickling (A) and with tickling (B). Nodes are represented by circles and edges as arrows that go from the play actor to the play receiver. The different colors of nodes indicate different modularity clusters. Nodes with solid double‐line outlines indicate adults; nodes with solid single‐line outlines indicate juvenile individuals and nodes with dashed outlines indicate immature subjects. Node size is based on weighted in‐degree centrality.

We then explored whether social bonding influenced tickling behavior. We found that the dyads characterized by a strong social bond spent significantly more time in tickling interactions on the total time spent playing compared to the dyads characterized by a weak social bond (Mann Whitney *U* = 33.000; *N*_strong_ = 6; *N*_weak_ = 35; *p* = 0.006; Figure 6). For the individuals who were involved in at least one play session, we found that individual clustering coefficients did not correlate between social play networks with and without tickling (*N*_individuals _= 10; *r* = –0.184; *p* = 0.611). Individuals cluster in a different way in the social networks of social play with and without tickling, as shown in Figure 7, based on modularity values. However, the main mother–infant clusters are preserved in the two networks. The social network showing all play sessions with and without tickling is shown in Figure S1.

## 4 Discussion

The results of this study show that bonobos share with humans some developmental and social features of tickling behavior. Our study on bonobos (*Pan paniscus*) suggests that basic elements of intersubjective abilities might have already been present in the *Homo‐Pan* last common ancestor, as similar features have been qualitatively reported for chimpanzees (*Pan troglodytes*) (Bard et al. 2014; Leavens and Bard 2016).

At the individual level, older individuals were most likely to tickle others and younger individuals were most likely to receive tickling. At the dyadic level, tickling was mostly directed from older to younger individuals. Moreover, tickling was mostly performed by the individuals who also initiated the play session. Finally, tickling was predominantly observed in dyads characterized by higher levels of affiliation, especially mother–infant dyads. We also observed that individuals clustered differently when they were involved in play session with or without tickling. It means that the individuals that are grouped together in the network of play with tickling based on the modularity values were not necessarily grouped together in the network of play without tickling. This suggests that play with tickling might serve a different function than play without it. All our predictions were thus validated. This study has been carried out in one captive bonobo group; further studies are needed to investigate whether our results are confirmed in other groups.

The effect of age—on the tickling actor and the receiver side—indicates that tickling was primarily performed by adults toward younger individuals. No tickling was performed on bonobos over 14, but we recorded one case in which an adult male was tickled by a younger, juvenile male. Although anecdotical, this observation suggests that bonobos continue to be ticklish during adulthood, as it happens in our species. Moreover, on the internet, it is possible to find several videos showing human caregivers tickling adult great apes that respond laughing (e.g., Kanzi the bonobo). Unfortunately, we did not observe play interactions among bonobo adults, and therefore it was not possible to investigate whether tickling interactions are present in adult–adult play. This feature, which would mirror what happens in our species, needs further investigation. The bonobo is a good model species to understand whether tickling is used during adult play, as they tend to maintain high levels of play also during adulthood (Palagi 2008).

The analysis of play invitations revealed that the initiator of the play session was also the one who performed more tickling, which suggests that play was specifically initiated to engage in tickling. Furthermore, the effect of social relationship indicates that tickling was most frequently expressed in pairs of individuals with highest affiliation levels, starting from mother–infant dyads. The social networks show that social play with tickling was not distributed as social play without tickling, thus suggesting that play tickling may solve a different function compared with other playful interactions. Therefore, the overall picture emerging from our study is that tickling may be used intentionally by adults on younger individuals with whom they share a close social bond.

As a whole, our results are in line with the qualitative observations carried out on chimpanzees (Bard et al. 2014; Leavens and Bard 2016) and support the idea that tickling is an infant‐directed behavior in nonhuman hominins, just as it is in humans. These findings may be linked with one hypothesized function of tickling: if tickling has evolved to develop combat skills during play‐fighting (Harris 2012), it might decrease during development as a consequence of the acquisition of such abilities and, therefore, be absent in mature individuals. To date, no study has tested if tickling sensitivity is preserved in adult great apes like it appears to be the case for adult humans (Harris 1999; Selden 2004). Nevertheless, the idea that tickling and its related response evolved to strengthen combat skills has received little support as most of the researchers are more prone to see tickling as a play behavior that evolved as a specific form of communication enhancing social bonding.

In line with this reasoning, strongly affiliated individuals spent significantly more time tickling each other compared with dyads characterized by weak affiliation levels. This finding supports the idea that tickling in bonobos, as it is in humans (Provine 2004; Rothbart 1973; Selden 2004), is a phenomenon that is adjusted depending on the social bond shared between actor and receiver. Indeed, in humans tickling can be perceived as pleasurable or aversive depending on the actor's familiarity with the receiver (Harris and Alvarado 2005; Harris 2012). This means that the receiver can recognize the affiliative relationship with the actor and respond accordingly. Because hormone rates appear to influence the play behaviors, it will be interesting to investigate their effects on the bonobo's responses to tickling. For example, in rats both oxytocin and glucocorticoids are involved in the modulation of social play (e.g., Papilloud et al. 2018; Vanderschuren, Achterberg, and Trezza 2016). However, an increase in oxytocin in humans and nonhuman species is usually associated with affiliative social behavior (e.g., Anacker and Beery 2013; Wittig et al. 2014), whereas glucocorticoids most commonly increase in stressful situations (e.g., Suarez‐Bregua, Guerreiro, and Rotllant 2018 *)*. In future studies, these hormones could be used as markers to assess the pleasurable or unpleasurable nature of tickling depending on actor's identity.

The tickling social network built in our study supports the idea that tickling in bonobos is a behavior that develops within the mother–infant pair, as it occurs in humans (Ishijima and Negayama 2017). Human and chimpanzee infants start requesting tickling to familiar individuals at the later stage of development, probably when they understand that the sensation that it causes can only be brought about by an external agent (Bard et al. 2014; Plooij 1978). Indeed, in human infants, tickling‐evoked laughter emerges around six‐7 months of age (Leuba 1941) along with the ability to recognize between self and others emotional states (Stevanovic and Koski 2018). Further investigation on tickling request in bonobos is welcome to better understand when in this ape the cognitive ability to distinguish between the self from the other, and between different others (familiar or not).

Tickling may have evolved as an emotional communicative pattern to reinforce mother–infant social bond (Harris 2012; Leuba 1941; Provine 2004). This may be possible because tickling induces laughter and its associated facial expression, namely the play face both in humans (Kurtz and Algoe 2015) and nonhuman apes (Demuru, Ferrari, and Palagi 2015). When the receiver responds to a perceived play face with another play face—a phenomenon known as rapid facial mimicry when the facial expression replication occurs within 1 s)—an emotional connection might be established (Preston and de Waal 2002). Such connection can take the form of emotional contagion, involving the transmission of a positive state from a subject to another, via the perception‐action mechanism (de Waal and Preston 2017). The mimicry of the play face that is triggered during tickling might induce emotional contagion and therefore reinforce social bonding.

In conclusion, tickling may indicate the presence of self‐other distinction and (depending on tickler familiarity) the ability to differentiate between different others, two building blocks of cognitive and socio‐emotional abilities related to intersubjectivity (Gärdenfors 2007). Because intraspecific tickling has been observed only in great apes and humans, probably in relation to complex socio‐cognitive communication, we suggest that it can be used as a behavioral marker to investigate the biological bases of intersubjectivity within the hominid family.

## Author Contributions

**Elisa Demuru:** data collection, supervision, conceptualization, methodology, formal analysis, data curation, writing–original draft, writing –review and editing. **Ilenia Montello:** video analysis, data curation, data analysis, writing–original draft. **Jean‐Pascal Guéry:** access to facilities and data collection facilitation. **François Pellegrino** and **Florence Levréro:** writing–review and editing. **Ivan Norscia:** conceptualization, methodology, data curation, writing–original draft, writing–review and editing, supervision.

## Ethics Statement

This study was purely observational. No approval was required from the authors' institution.

## Conflicts of Interest

The authors declare no conflicts of interest.

## Supporting information


**Supplementary Figure 1** – General social network of social play with and without tickling. Nodes are represented by circles and edges as arrows that go from the play actor to the play receiver. The different colors of nodes indicate different modularity clusters. Nodes with solid double‐line outlines indicate adults; nodes with solid single‐line outlines indicate juvenile individuals, and nodes with dashed outlines indicate immature subjects. Node size is based on weighted in‐degree centrality.

**Supplementary Video**. Example video of a sequence of tickling play between a bonobo mother and her infant. Note: the mother's choice of a ticklish area of the infant's body (i.e. armpit), the repetitive nature of the action; the infant's behavioural reactions (i.e. wriggling, pulling, sheltering), facial expressions (i.e. play face and full play face) and the active search for a visual contact.

Supplementary Table 1. Composition of the bonobo colony housed at *La Vallée des Singes* during the data collection period.

## Data Availability

Data are available as supplementary material.
