# Supplementary figures and images for: Tackling Hominin Tickling: Bonobos Share the Social Features and Developmental Dynamics of Play Tickling With Humans
Source: Am J Primatol. 2025 Jan 15;87(1):e23723. doi: 10.1002/ajp.23723 (PMC11734381; doi:10.1002/ajp.23723)

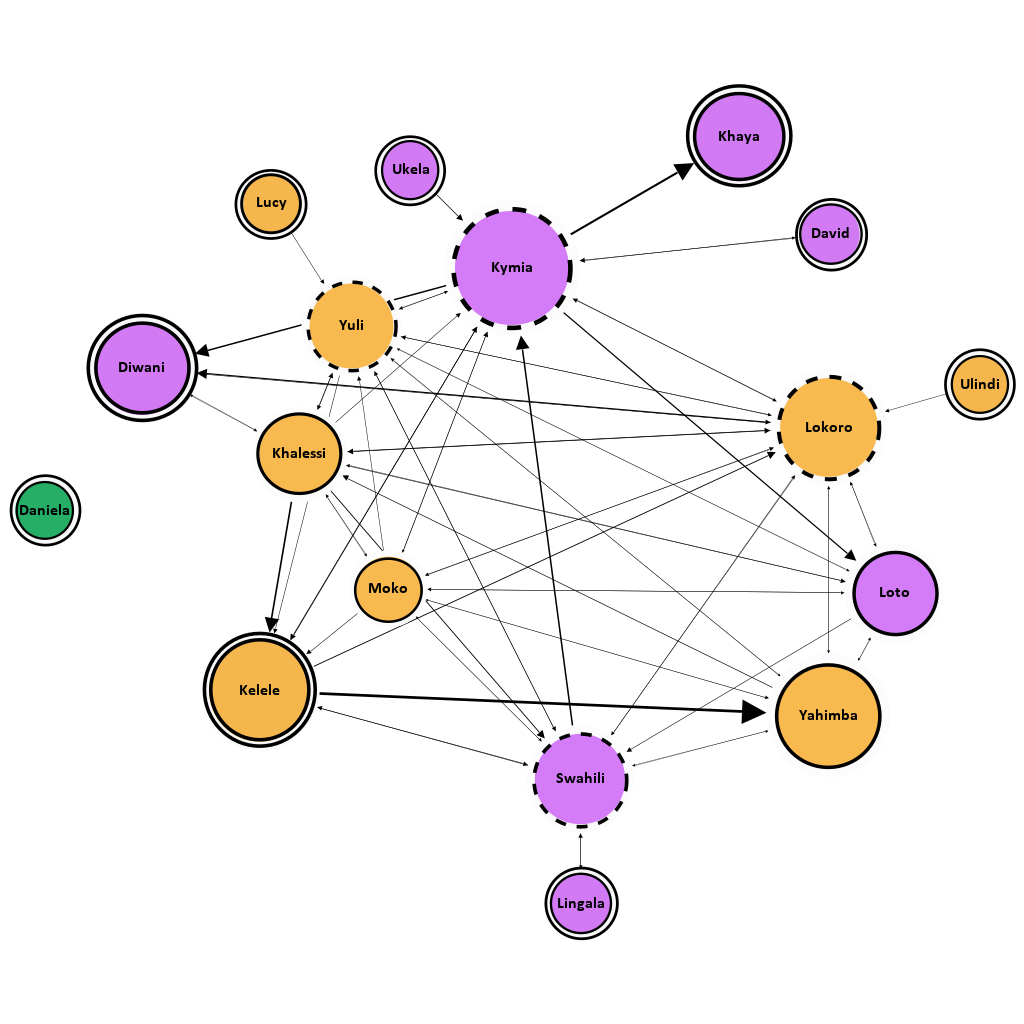

Supplement: Supplementary file 1 — Supplementary Figure 1 – General social network of social play with and without tickling. Nodes are represented by circles and edges as arrows that go from the play actor to the play receiver. The different colors of nodes indicate different modularity clusters. Nodes with solid double‐line outlines indicate adults; nodes with solid single‐line outlines indicate juvenile individuals, and nodes with dashed outlines indicate immature subjects. Node size is based on weighted in‐degree centrality. [file AJP-87-e23723-s002.png]
