# Supplementary material for: Tackling Hominin Tickling: Bonobos Share the Social Features and Developmental Dynamics of Play Tickling With Humans
Source: Am J Primatol. 2025 Jan 15;87(1):e23723. doi: 10.1002/ajp.23723 (PMC11734381; doi:10.1002/ajp.23723)
Supplement: Supplementary file 3 — Supplementary Table 1. Composition of the bonobo colony housed at La Vallée des Singes during the data collection period. [file AJP-87-e23723-s001.docx]

| Name | Sex | Age | Age class | Kinship | Role during tickling interactions |
| --- | --- | --- | --- | --- | --- |
| Daniela | F | 50 | Adult | David’s and Diwani’s mother | Only actor. |
| Ukela | F | 33 | Adult | Kymia’s and Moko’s mother | Only actor. |
| Ulindi | F | 25 | Adult | Loto and Lokoro’s mother | Only actor. |
| Diwani | M | 22 | Adult | Daniela’s son | Only actor. |
| David | M | 17 | Adult | Daniela’s son | Never performed of received it. |
| Khaya | F | 17 | Adult | Khalessi’s mother | Never performed of received it. |
| Lingala | F | 15 | Adult | Swahili’s mother | Only actor. |
| Lucy | F | 15 | Adult | Yuli’s mother | Only actor. |
| Kelele | M | 14 | Adult | No kinship with other group members | Actor and receiver. |
| Loto | M | 9 | Juvenile | Ulindi’s son | Actor and receiver. |
| Yahimba | F | 9 | Juvenile | No kinship with other group members | Actor and receiver. |
| Khalessi | F | 6 | Juvenile | Khaya’s daughter | Actor and receiver. |
| Moko | M | 6 | Juvenile | Ukela’s son | Actor and receiver. |
| Swahili | F | 4 | Infant | Lingala’s daughter | Actor and receiver. |
| Yuli | F | 4 | Infant | Lucy’s daughter | Actor and receiver. |
| Lokoro | M | 3 | Infant | Ulindi’s son | Actor and receiver. |
| Kymia | F | 1 | Infant | Ukela’s daughter | Only receiver. |

**Supplementary Table**

Individuals composing the study group and their role during tickling interactions
